# Methylation changes associated with early maturation stages in the Atlantic salmon

**DOI:** 10.1186/1471-2156-12-86

**Published:** 2011-10-07

**Authors:** Paloma Morán, Andrés Pérez-Figueroa

**Affiliations:** 1 Dpto. Bioquímica, Xenética e Inmunoloxía. Faculta de Biología. Universidade de Vigo. Vigo 36310. Spain

## Abstract

**Background:**

Early maturation in the Atlantic salmon is an interesting subject for numerous research lines. Prior to sea migration, parr can reach sexual maturation and successfully fertilize adult female eggs during the reproductive season. These individuals are known as precocious parr, mature parr or "sneakers". Reasons for early maturation are unknown and this transitory stage is usually considered to be a threshold trait. Here, we compare methylation patterns between mature and immature salmon parr from two different rivers in order to infer if such methylation differences may be related to their maturation condition. First we analyzed genetic differences between rivers by means of AFLPs. Then, we compared the DNA methylation differences between mature and immature parrs, using a Methylation-Sensitive Amplified Polymorphism (MSAP), which is a modification of the AFLPs method by making use of the differential sensitivity of a pair of restriction enzymes isoschizomeres to cytosine methylation. The tissues essayed included brain, liver and gonads.

**Results:**

AFLPs statistical analysis showed that there was no significant differentiation between rivers or a significant differentiation between maturation states in each river. MSAP statistical analysis showed that among the three tissues sampled, the gonads had the highest number of significant single-locus variation among populations with 74 loci followed by brain with 70 and finally liver with only 12. Principal components analysis (PCA) of the MSAP profiles revealed different profiles among different tissues (liver, brain and testis) clearly separating maturation states in the testis tissue when compared to the liver.

**Conclusions:**

Our results reveal that genetically-similar mature and immature salmon parr present high levels of DNA methylation variation in two of the three analyzed tissues. We hypothesize that early maturation may be mostly mediated by epigenetic processes rather than by genetic differences between parrs. To our knowledge this is the first study that attempt to link phenotypic plasticity in salmonids and epigenetic changes.

## Background

Atlantic salmon populations are anadromous with the only exception of those that inhabit rivers or lakes where there are physical impediments to seaward migration [1]. Eggs develop over winter and hatch in the following spring. After hatching, the fry stay for one to several years in the river and become parr. During spring-early summer, immature parrs undergo parr-smolt transformation and migrate downstream to the sea. After spending several years in the sea, the adults return to spawn in their native river in November-December [2]. Alternatively, during the first or second year in freshwater Atlantic salmon male parr can precociously mature.

During the reproductive season, mature male parr compete with the larger anadromous males for access to anadromous females during spawning and are able to fertilize high proportions of eggs [3-9] and as consequence, the effective size of Atlantic salmon populations, increases [10].

Early maturation is observed in mainly all the populations and their evolutionary advantages have been extensively reviewed [11,12]. It has been observed that the incidence of mature male parr varied between rivers and even between seasons for a given salmon population. Moreover, a negative relationship between male parr maturation rates and geographical latitude for both American and European populations has also been found [13].

Reasons for early maturation are unknown. Several investigations suggest that maturity age is genetically determined [2,12,14] and significantly associated to growth rate during the first or second year of their life. Accordingly, Piché et al. [14] have hypothesized that maturity in the male Atlantic salmon is a threshold trait and therefore, maturation is dependent upon the attainment of a critical growth rate and body size. Because of the genetic variability associated with population growth rates, the turning on points for early maturation in each population will partly depend on the distribution of the individual growth rates. However, other studies have found no direct evidence of a link between the incidence of precocious maturation and early life characteristics [15]. Regardless of the causes of early maturation, it has been clearly demonstrated that it is a transitory state. Mature parr will become an anadromous male in the following season and, as an adult male, it will return to its natal river to spawn after one or two years of growth in the sea [16].

Since those differences in life cycle stages, such as growing and maturation, imply differences in gene expression, in recent years many researchers have paid more attention to the study of transcriptome, using a wide spectrum of techniques such as microarrays and quantitative PCR [17]. Early maturation in parr has been the subject of an extensive study comparing gene expression in mature and immature parrs [18]. This study compared changes in gene expression in brain and testes revealing greater changes in testes than in brain allowing the identification of genes that are up- and down-regulated in mature parr testes.

Gene regulation involves different mechanisms; any step of gene expression can be modulated from DNA modifications to mRNA degradation. A common method of gene silencing is DNA methylation (see [19] and references therein). It consists in a chemical modification of the genomic DNA that involves the binding of a methyl group to a nucleotide, often the 5' carbon of the cytosine pyrimidine ring. DNA is typically methylated by methyltransferase enzymes on cytosine nucleotides in a CpG dinucleotide sequence and, as consequence methylated DNA sequences are transcriptionally inactive. DNA methylation has been well characterized in mammals, where it is restricted to CpG dinucleotides and in plants where the cytosine can be methylated at CpG, CpNpG, and CpNpN sites. In other species DNA methylation is poorly characterized (reviewed by [20]).

DNA methylation of cytosine residue is, together with chromatin remodeling through chemical modification and regulatory processes mediated by small RNA molecules, part of the epigenetic process [19]. Epigenetics has been redefined as heritable changes in gene expression that cannot be tied up to genetic variation [21].

Changes in the methylation pattern might not be due to heredity but instead be stimulated by genomic stress and environmental changes [22]. A wide variety of published literature could be found about modulation and regulation of gene expression due to methylation in plants [23] and humans, especially those relating methylation to cancer [24]. Little attention, however, has been paid to the extent to which methylation could affect gene regulation in other eukaryotic species. Methylation studies in fishes are rather scarce with most focus on zebrafish [25], although there are some studies in medaka [26] and one in rainbow trout [27].

In the present study, we compared the genetic and epigenetic (specifically, DNA methylation) changes between mature and immature male parr. Juvenile salmons from two different populations were analyzed in order to test the repeatability of the results. Genetic variations were screened by means of the amplified fragment length polymorphism (AFLP) technique and epigenetic differences between mature and immature male parr were surveyed, using a Methylation-Sensitive Amplified Polymorphism (MSAP) in three different tissues (brain, liver and testis). MASP is the methylation sensitive modification of the AFLP technique. *Mse*I is replaced with the isoschizomers *Hpa*II and *Mse*I in parallel reactions. Each restriction enzyme recognizes the sequence CCGG but differs in its sensitivity to DNA methylation at the inner cytosine [28]. This technique has proved useful to uncover epigenetic variability in plant and animals species [29,30].

Our ultimate aim was to compare genetic and methylation patterns to infer if methylation differences could be related to early maturation.

## Results

### Methylation-susceptible loci

The three primer combinations assayed with the MSAP analysis produced a total of 655, 669 and 591 bands (loci) in the brain, liver and gonad samples, respectively. Table 1 shows the number of methylation-susceptible loci (MSL) and which ones were polymorphic (i.e. at least two occurrences for every state). Only these polymorphic MSL (around one third of the total number of loci obtained) were used for the analyses.

**Table 1:** Epigenetic variation

Tissue	**Number of MSL**^**a**^	**Number of polymorphic MSL**^**b**^	**% significant single-locus variation among populations **^**c**^	**Diversity**^**d**^
Brain	429 (65.5%)	262	27.7 (*q *= 0.093)	0.50 (0.15)
Liver	418 (62.5%)	218	5.5 (*q *= 0.230)	0.48 (0.15)
Gonad	429 (72.6%)	206	35.9 (*q *= 0.093)	0.50 (0.15)

^a ^Methylation-susceptible loci.

^b ^A methylation-susceptible loci was considered polymorphic when at least two individuals showed the non-methylated state.

^c ^% of the significant single-locus homogeneity tests (χ^2^) after multiple test correction with sequential combined probability test of Fisher [47]□. *q-values *are given between brackets providing the expected proportion of false positives.

^d ^Average Shannon's Diversity Index (±SD).

Data transformation, where bands absent from both *EcoR*I-*Hpa*II and *EcoR*I-*Msp*I products represent undefined scores, yield a total of 25.9%, 31.7% and 32.1% of such missing values for brain, liver and gonad respectively.

Of the three tissues sampled, the gonad had the highest number of significant single-locus variation among populations (see Table 1) with 74 loci (after multiple test correction), followed by brain with 70 and then liver with only 12. The q-values estimated for the homogeneity tests suggested that among those significant loci 7,7 and 4 false positives should be expected in gonad, brain and liver, respectively. On the other hand, the three tissues were equally diverse, as measured by Shannon's Diversity Index (Table 1)

### Genetic differentiation

**Figure 1:**
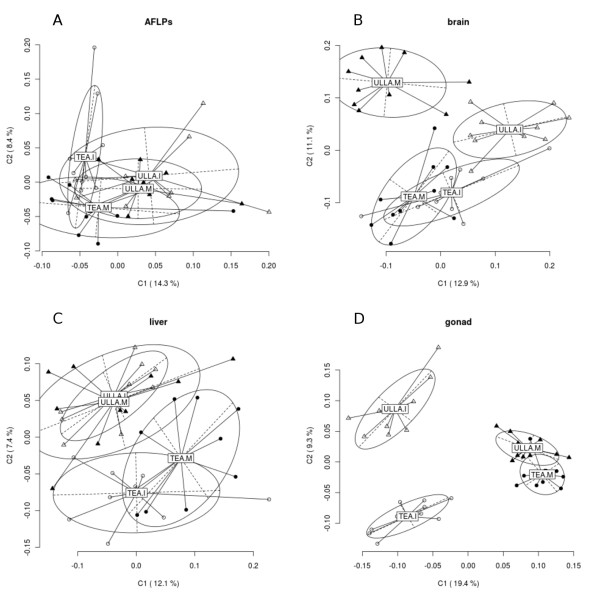
**Principal Coordinates Analysis (PCoA) results for genetic (AFLPs, panel A) and epigenetic (brain, liver and gonad, panel B, C and D respectively) differentiation between rivers and maturity status**. The first two coordinates (C1 and C2) are shown with the percentage of variance explained by them between parentheses. Circles represent individuals from Tea river and triangles from Ulla river. Open symbols represent immature individuals while filled symbols represent mature. Population labels show the centroid for the points cloud in each population. Ellipses represent the dispersion of those points around their center. The long axis of the ellipse shows the direction of maximum dispersion and the short axis, the direction of minimum dispersion.

A total of 127 standard AFLP fragments were obtained. χ^2 ^tests for population heterogeneity yielded, after multiple test corrections, a total of 6 loci significantly (*q *= 0.084) different between populations. AMOVA-based population differentiation results are given in Table 2. The levels of differentiation were measured as Φ_ST _(Excoffier et al. 1992). AMOVA showed that there was no significant differentiation between rivers or between maturation stages in each river. PCoA also showed (Figure 1.A) that there is no significant differentiation for both comparisons

**Table 2:** Genetic and epigenetic multiloci differentiation between rivers and maturation states

	Sample	**Φ**_**ST **_**between rivers**	**Φ**_**ST **_**between maturation states**	
			
			Tea	Ulla
AFLPs	Liver	-0.01 (*P *= 0.33)	0.03 (*P *= 0.33)	0.02 (*P *= 0.66)

MSAP	Brain	0.05 (*P *= 0.33)	**0.07 (*P *< 0.0001)**	**0.25 (*P *< 0.0001)**
	Liver	0.07 (*P *= 0.34)	0.04 (*P *= 0.33)	0.01 (*P *= 0.33)
	Gonad	-0.07 (*P *= 0.66)	0.41 (*P *= 0.33)	**0.42 (*P *< 0.0001)**

AMOVA results following [46]. *P*-values were derived from a random permutation test with 10000 permutations. Bold values show significant differentiation (*P *< 0.05).

### Epigenetic multi-locus differentiation

AMOVA showed no significant differentiation between rivers or maturation states when liver samples were analyzed for MSAP (Table 2)

However, there was a consistent pattern of differentiation between maturation states in the other two tissues. In the brain, significant differences were detected when mature and immature male parr were compared and higher values were obtained for river Ulla than for river Tea. It is noticeable that in the gonad the differentiation between maturation stages was especially high (Φ_ST _> 0.40) for both rivers.

The PCoA MSAP variation revealed different levels of epigenetic variation between rivers and maturity status. The output plots of the two first principal coordinates are showed in Figure 1. In the case of liver (Figure 1.C) only some differentiation between rivers could be observed, mainly along the second coordinate (7.4% of variance explained), whereas no clear pattern emerged for the maturation stages. Second Coordinate also separated the brain samples (Figure 1.B) into two groups according to their river origin (11.1% of variance explained), whereas the first coordinate allowed the distinction between maturation stages but clustered the parr from the two rivers. Finally, mature parr gonad (figure 1.D) was clearly separated from immature parr one along the first coordinate (19.4% of variance explained). Furthermore, a slight differentiation between rivers along the second axis (9.4% of variance explained), especially for immature individuals, could also be observed. The overall clustering patterns confirm AMOVA results.

## Discussion

We have explored both genetic and epigenetic variation in mature and immature individuals of two different salmon male parr populations and have shown that individuals with similar genetic profiles present divergent epigenetic profiles in different tissues. Further, we show that these differences are correlated with their maturation stage.

The amplified fragment length polymorphism (AFLP) analysis revealed a high level of genetic diversity in salmon. The comparison between AFLPs patterns of rivers Ulla and Tea revealed slight, although not statistically significant, differences between rivers. Overall, salmon populations show considerable reproductive isolation which has allowed the development of local adaptations, and as a result, significant genetic differences were observed between populations from different rivers [31]. Although there are no previous reported population analysis using AFLPs in the Atlantic salmon, studies in brown trout [32] reveal high levels of polymorphic loci (up to 61%) suggesting that some differences could also be expected in the salmon populations due to the high number of polymorphic loci that can be detected using this wide scan genome method. Furthermore, as anticipated, no genetic differentiation was observed between mature and immature male parr at each of the two analyzed populations. To date, differences between mature and mature parr have been observed in gene expression but not in neutral genetic markers (see [33] and references therein)

The lack of genetic divergence in both populations prompted us to look into the possible epigenetic divergence between mature and immature parrs. In a previous study, we developed a strategy based on MSAP profile analyses and epigenetic diagnostic markers to enable us to distinguish and to separate different tissues of Atlantic salmon and veal products with identical genetic information [34]. Taking into account our findings and those derived from gene expression analysis by Guiry et al. [18], we examined the methylation profiles in the liver, brain and gonad tissues, providing strong evidence that methylation levels are different in these tissues. For each tissue analyzed the PCoA of the MSAP profiles revealed different levels of separation according to their maturation stages. Clearer separation was seen between the two maturation stages for gonad tissue, but less obvious for brain tissue, and very little distinction for liver tissue. In contrast, the AMOVA test showed significant differentiation between maturation stages for brain tissue in both rivers, in river Ulla only for gonad tissue but, high differentiation (Φ_ST _> 0.4) is observed in both rivers. No differentiation was observed in liver tissue.

Overall, these results are in agreement with the differences in gene expression observed in brain and testes in mature male parr previously observed [18]. According to this study, small expression changes in brain but pronounced changes in testes during the process of precocious sexual maturation were recorded. We should emphasize that not all methylation differences between mature and immature parr can be exclusively interpreted as differences due to maturation stage. Other physiological changes such as smoltification may occur simultaneously [35] obscuring the underlined processes involved in early maturation. Nevertheless, we hypothesize that early maturation could be mainly mediated by epigenetic processes rather than by genetic differences between parrs. A crucial aspect is the strong differentiation found in gonad that could be difficult to explain without this hypothesis.

Methylation of DNA is one of the major epigenetic markers that affect gene expression either directly or indirectly [36]. It is a dynamic process that takes place throughout the course of development, and at the same time is an important target for environmental modification (see [22] and references therein) providing an additional source of variation that could mediate the relationship between genotype and internal and external environmental factors. This rationale circumvents genotypic differences between individuals and encompasses internal and external factors such as growth rate and environmental conditions when trying to explain phenotypic plasticity. In this context, the inter-annual variations in percentages of early maturation for any given population could be easily explained through an environmental-mediated DNA methylation mechanism. Our findings indicate that, in addition to genetic information, the epigenetic component of salmon genome could play an important role in early maturation.

To our knowledge this is the first study that attempts to link phenotypic plasticity and epigenetic changes in salmonids, despite the recent interest on the topic (see review by [37]).

## Conclusions

In many species, phenotypic differences can be explained as methylation differences between individuals. For example, it has been shown that artificial demethylation can alter the phenotypic plasticity patterns of *Arabidopsis thaliana*, as well as the amount of observed phenotypic variation among plant individuals and genotype means [38]. Our results reveal that genetically-identical mature and immature salmon parr present high levels of DNA methylation differentiation in two of the three analyzes tissues. We, therefore, hypothesize that early maturation could be mostly mediated by epigenetic processes rather than by genetic differences between parrs. The study of methylation patterns could have a profound impact in ecological and evolutionary studies [22]. We argue that epigenetic studies should be considered in further studies trying to explain the phenotypic plasticity in salmon and other related species.

## Methods

### Experimental design

Methylation patterns in mature male parr and immature male par were compared as follows: during November 2008 wild native salmon spawners were caught in Rivers Ulla and Tea and transported to the nearby Carballedo salmon hatchery until stripping. Incubation post-fertilization temperature was approximately 5 ± 1°C. After the first feeding, each population was reared outdoors in identical, separate fibreglass tanks and fed in excess on commercial food pellets. The hatchery rearing troughs were uniform in size, structure and water quality Average temperature during this phase of the rearing cycle was 11.5°C. In November 2009, ten mature male parr were selected by squeezing gently until milt was expressed. In addition, 15 parrs from river Ulla and 18 parrs from river Tea were euthanized using MS-222 (Sigma). Ten immature male parrs from the offspring of each river were selected by gonad visual inspection under magnifying glass. Female parr were discarded. Size average of the selected parr was 83.1 ± 93 mm. The whole brain and tissue samples of liver and gonads (testes) of selected male parrs were extracted. The rationale behind the analysis of two different populations reared under the same conditions was to determine whether the results are not associated with any given sample and could be extrapolated to others populations. The experiment was performed with the approval of the University Ethics committee.

### DNA isolation and AFLP genotyping

DNA was extracted from tissue samples using the NucleoSpin^® ^Tissue Kit BD Biosciences. DNA quality was verified by electrophoresis on 1% agarose gels. After DNA quantification using a Nanodrop 1000 spectrophotometer (Thermo Fisher Scientific), samples were normalized to 100 ng μl^-1^.

Genetic differences between rivers were measured comparing AFLPs profiles. AFLP methodology represented a modified version of [39]. For each individual, 50 ng of DNA were digested and ligated using 5 U of *EcoR*I and 3 U of *Mse*I (New England Biolabs), 5 pmol *EcoR*I adaptor, 50 pmol *Mse*I adaptor and 0.4 U of T4 DNA ligase (Roche) in 20 μl total volume of 1X NEB buffer #2 (50 mM NaCl; 10 mM Tris-HCl; 10 mM MgCl_2_; 1 mM DTT; pH 7.9) and 2X ligation buffer (Roche) (660 mM Tris-HCl; 50 mM MgCl_2_; 50 mM DTT; 10 mM ATP; pH 7.5) supplemented with 2.5 μg of BSA for 2 h at 37°C.

Preselective PCR reactions were then performed in 4 μl of 1:10 ligation dilution in 20 μl volumes containing 2.5 mM of MgCl_2_, 187.5 μM of each dNTP, 20 pmol of *Eco*RI-A and *Mse*I-C preselective primers and 1 U of Taq polymerase (Bioline) in 1X PCR buffer (Bioline). PCR conditions for preselective PCR were as follows: 72°C for 2 min, 20 cycles of 94°C for 20 s, 56°C for 30 s, 72°C for 2 min, and a final step of 60°C for 30 min. Selective PCR reactions were performed in 4 μl of 1:10 preselective PCR dilution in 20 μl volumes containing 2.5 mM of MgCl_2_, 187.5 μM of each dNTP, 8.3 pmol of *EcoR*I-ACT and *Mse*I-CAC selective primers and 1 U of Taq polymerase in 1X PCR buffer. Cycling conditions for selective PCR were as follows: 94°C for 2 min, 10 cycles of 94°C for 20 s, 66°C (decreasing by 1°C each cycle) for 30 s, and 72°C for 2 min, followed by 20 cycles of 94°C for 20 s, 56°C for 30 s, and 72°C for 2 min, ending with 60°C for 30 min.

### MSAP genotyping

Methylation differences between maturation stages were measured comparing MSAP profiles. MSAP methodology represented a modified version of [40] and [41] and is basically the AFLPs protocol previously described replacing the 3 U of *Mse*I in the digestion-ligation step for 1U of *Hpa*II or *Msp*I (New England Biolabs), and the *Mse*I adaptor for the *Hpa*II adaptor. In the successive steps *Mse*I primers were replaced with *Hpa*II primers as detailed in Table 3. *Hpa*II and *Msp*I are isoschizomers recognize the same sequence (5*'*-CCGG) but differ in their sensitivity to DNA methylation. Comparison of the two profiles for each individual allowed the assessment of the methylation state of the restriction sites. Methylated CpG are restricted by *Msp*I only, hemimethylated CpCpG sites are restricted by *Hpa*II only (the restriction enzyme database (*rebase.neb.com/rebase/rebase.html)*). Sites that are hypermethylated (i.e., both at the internal and external Cs), and sites that are fully methylated at the external Cs (i.e., on both strands) are not cut by either enzyme, whereas sites that are free from methylation are restricted by both.

**Table 3:** MSAP primer sequences used in this work

Oligo name	Function	Sequence
Ad.*Hpa*II/*Msp*I Rv	Adaptor	GACGATGAGTCTAGAA
Ad.*Hpa*II/*Msp*I Fw	Adaptor	CGTTCTAGACTCATC
Ad.*Eco*RI Rv	Adaptor	AATTGGTACGCAGTCTAC
Ad.*Eco*RI Fw	Adaptor	CTCGTAGACTGCGTACC
Pre. *Eco*RI	Preselective primer	GACTGCGTACCAATTCA
Pre. *Hpa*II/*Msp*I	Preselective primer	GATGAGTCTAGAACGGT
*Eco*RI + ACT	Selective primer	GACTGCGTACCAATTCACT
*Eco*RI + AAG	Selective primer	GACTGCGTACCAATTCAAG
*Hpa*II + TAC	Selective primer	GATGAGTCTAGAACGGTAC
*Hpa*II + TC	Selective primer	GATGAGTCTAGAACGGTC

A total of 3 primer combinations ( *EcoR*I-AAG- *Hpa*II -TC, *EcoR*I -ACT- *Hpa*II -TC and *EcoR*I -AAG- *Hpa*II -TAC) E were used for selective amplifications (Table 3). *Hpa*II primers were end labelled using a 6-FAM reporter molecule. PCR products were loaded simultaneously with a GeneScan 500 ROX size standard into an ABI Prism 310 Genetic Analyzer (Applied Biosystems). Fragment analysis and AFLP scoring was performed using GeneMapper v.3.7 software (Applied Biosystems). DNA fragments less than 100 bp in length, longer than 500 bp or less than 70 RFU (Relative Fluorescent Units) were excluded from the analysis due to low levels of reproducibility. Four random individuals were chosen to determine the repeatability of the AFLP protocol and scoring method. Three replicates were performed for each one of the individuals and the repeatability, obtained by averaging for all primer sets used, was 94.7% ± 0.5.

### Data analysis

MSAP data for all primer combinations were mixed for each tissue. We divided the samples into four populations considering the river of origin and the maturity status (M, mature; I, Inmature): ULLA.M, ULLA.I TEA.M and TEA.I.

Analyses of MSAP results were performed following [42]. For every sample and particular fragment, we first determined whether the fragment was: (1) present in both *EcoR*I-*Hpa*II and *EcoRI*-*Msp*I products, denoting a nonmethylated state; (2) absent from both *EcoR*I-*Hpa*II and *EcoR*I-*Msp*I products, being an uninformative state as it could be caused by either fragment absence or hyper-methylation; or (3) present only in either *EcoR*I-*Hpa*II or *EcoR*I-*Msp*I products, corresponding to a methylated state. Individual fragments were classified as either 'methylation-susceptible' or 'non-methylated', depending on whether the observed proportion of discordant *Hpa*II-*Msp*I scores suggestive of methylation (i.e. number of individuals with contrasting *Hpa*II-*Msp*I scores for the fragment divided by total number of individuals assayed) exceeded a 5% threshold, rounding the repeatability value obtained before. Non-methylated loci were scored as dominant binary markers, as usually done for AFLP markers (1 and 0, for fragment presence and absence, respectively). Instances of discordant *Hpa*II-*Msp*I scores in non-methylated fragments were resolved according to fragment presence. Methylation-susceptible fragments were scored as if the methylated state was an imperfectly assessed dominant marker: 1 for the methylated state, 0 for the non-methylated state and unknown (i.e. score missing) for uninformative state [42].

The amount of genetic variation was estimated using by the Shannon diversity index (*S*), which was calculated for each locus by the formula *S *= -∑*P*_*i *_log_e_(*P*_*i*_) where *P*_*i *_is the frequency of the presence or absence of the band (i = 1, 2). The mean diversity was estimated by an average of index values over individual loci.

Statistical analysis of MSAP results followed a band-based strategy [43]. Genetic and epigenetic differentiation was assessed with principal components analysis (PCA) performed with the package ade4 [44,45]. Single-locus and multilocus epigenetic population differentiation were tested using χ^2 ^tests for population heterogeneity in methylation frequency using POPGENE [46] and analyses of molecular variance (AMOVA; [47]) using the package ade4, respectively. Given the large number of χ^2 ^tests we applied a multiple test correction with sequential combined probability test of Fisher [48] using the SGOF+ software ([49], http://webs.uvigo.es/acraaj/SGoF.htm). This software also provided an estimation of the *q-values *linked to each test, i.e. the expected proportion of false positives incurred if we considered a given test significant [50], by estimating the proportion of true null hypotheses following the standard deviation proportional bounding method [51].

## Authors' contributions

PM conceived, designed the study, performed the laboratory experiments and wrote the manuscript; APF performed the statistical analyses and helped with the draft of the manuscript. All authors read and approved the final document.
